# Introduction of quorum sensing elements into bacterial bioreporter circuits enhances explosives’ detection capabilities

**DOI:** 10.1002/elsc.202100134

**Published:** 2022-03-02

**Authors:** Etai Shpigel, Shiri Nathansohn, Anat Glozman, Rachel Rosen, Benjamin Shemer, Sharon Yagur‐Kroll, Tal Elad, Shimshon Belkin

**Affiliations:** ^1^ Department of Plant and Environmental Sciences The Alexander Silberman Institute of Life Sciences The Hebrew University of Jerusalem Jerusalem Israel

**Keywords:** 2,4‐dinitrotoluene (DNT), 2,4,6‐trinitrotoluene (TNT), bioluminescence, bioreporter, biosensor, explosives, quorum sensing

## Abstract

A possible solution for the standoff detection of buried landmines is based on the use of microbial bioreporters, genetically engineered to emit a remotely detectable optical signal in response to trace amounts of explosives’ signature chemicals, mostly 2,4‐dinitrotoluene (DNT). Previously developed DNT sensor strains were based on the fusion of a DNT‐inducible gene promoter to a reporting element, either a fluorescent protein gene or a bacterial bioluminescence gene cassette. In the present study, a different approach was used: the DNT‐inducible promoter activates, in *Escherichia coli*, the quorum‐sensing *luxI* and *luxR* genes of *Aliivibrio fischeri*. N‐Acyl homoserine lactone (AHL), synthesized by LuxI, combines with LuxR and activates the bioluminescence reporter genes. The resulting bioreporter displayed a dose‐dependent luminescent signal in the presence of DNT. Performance of the sensor strain was further enhanced by manipulation of the sensing element (combining the *E. coli* DNT‐inducible *azoR* and *yqjF* gene promoters), by replacing the luminescence gene cassette of *Photorhabdus luminescens luxCDABE* with *A. fischeri luxCDABEG*, and by introducing two mutations, *eutE* and *ygdD*, into the host strain. DNT detection sensitivity of the final bioreporter was over 340‐fold higher than the original construct.

**Abbreviations**
DNT: 2,4‐dinitrotoluene
GFP: green fluorescent protein
QS: quorum sensing
QSB: quorum‐sensing bioreporter
TNT: 2,4,6‐trinitrotoluene

## 1 INTRODUCTION

The prevalence of explosive remnants from past conflicts is a global problem that claims numerous victims each year. A crucial difficulty in the demining of large areas is the lack of a reliable, effective, and safe means to pinpoint the exact location of buried landmines and other explosive devices. Current methodologies mostly require the use of hand‐held metal detectors, focused on the detection of the mine's metal casing, employed by skilled operators in immediate proximity to the buried landmines. This method is not only hazardous to the involved personnel, but also highly inefficient, mostly due the inability to detect nonmetallic objects and to the extremely high false‐positive detection rate [1]. Several alternative methods have been developed, targeting the explosive vapors emitted from landmines rather than their metallic signature, including the use of dogs or rats. These methods are extremely expensive and time consuming, suffer from low consistency, and still require the presence of personnel on site. Analytical chemistry, which offers accurate and sensitive detection techniques for diverse explosives, necessitates nonportable and costly analytical devices, and – most important – requires the collection of a very large number of samples at the minefield. While some portable variants of these instruments exist [2], they suffer from a significantly reduced sensitivity and cannot effectively detect localized vapor signatures over large areas. There is thus an acute need for an alternative approach, which would combine a high detection efficiency with a remote detection technology.

One such alternative involves the use of explosive‐sensing microbial bioreporter strains. These are genetically engineered microorganisms, harboring a molecular fusion between a sensing element (often a gene promoter) induced by the target compound, to a reporting element that produces an optical signal that can be detected and quantified from a distance. These sensor strains are characterized by a rapid response, portability, field applicability, and low‐cost; coupled to a suitable optical imaging system, they may serve as a viable alternative to current detection technologies. Using this approach, we have previously demonstrated [3, 4] the remote detection of buried landmines. The bioreporter employed was an *Escherichia coli* strain, harboring a plasmid‐borne fusion of the *yqjF* gene promoter to the green fluorescent protein gene GFPmut2, immobilized in calcium alginate beads. Excitation of the fluorescent signal and its emission were conducted from a distance of ca 20 m. The *yqjF* gene promoter employed as a sensing element was previously shown [5] to be induced by 2,4,6‐trinitrotoluene (TNT), the most common landmine explosive, as well as by its manufacturing byproduct 2,4‐dinitrotoluene (DNT). The latter compound, due to its higher volatility, is considered a more suitable tracer for a landmine's presence [6, 7, 8].

**PRACTICAL APPLICATION**

The detection of buried landmines and other explosive devices requires the presence of personnel on the minefield, as there is no viable technology for the standoff detection of these deadly devices. One of the solutions proposed in answer to this need is to employ genetically engineered microorganisms, “tailored” to emit an optical signal in the response to the traces of explosives’ vapors emanating from the mines, accumulating in the soil above them. The bioluminescent explosive sensor strains designed, constructed and tested in the present article constitute a step forward in the effort to realize such a remote detection scheme.

Following a series of molecular manipulations and performance optimizations, the *yqjF* gene promoter was also fused to a bacterial bioluminescence *luxCDABE* gene cassette, yielding new bioluminescent explosives’ sensor strains [5, 9, 10]. Random mutagenesis of *the yqjF* promoter region has led to a significant enhancement of the reporter's performance in terms of signal intensity, response time and detection threshold [9]. Additional improvements were introduced by the selection of beneficial mutations in the host strain [11], “DNA shuffling” with an additional DNT/TNT‐responsive promoter, *azoR* [12], by optimization of the alginate encapsulation matrix, and by shifting to *luxCDABEG* gene cassettes from different origins [14].

In this communication we investigate an alternative to the simplistic promoter‐reporter fusion design: the introduction of quorum sensing (QS) elements as means of signal amplification. This natural microbial mechanism, originally described in bioluminescent bacteria as “autoinduction” [15], is involved in diverse processes such as symbiosis, virulence, competence, conjugation, antibiotic production, motility, sporulation, and biofilm formation. The accumulation of a small membrane‐permeable molecule promotes the activation of specific genes in response to changes in the density of the microbial community. This allows bacteria to function in unison and respond efficiently to external conditions [16, 17, 18, 19, 20].

**FIGURE 1:**
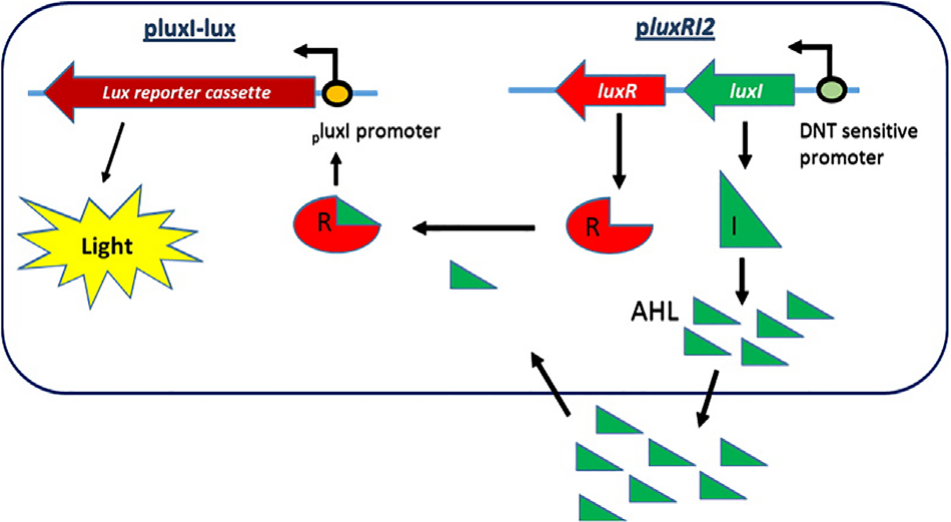
Schematic illustration of the two‐plasmid DNT‐responsive QS system

The QS system of the marine luminescent bacterium *Aliivibrio fischeri* contains two regulatory genes, *luxI* and *luxR*. The former encodes acyl‐homoserine‐lactone synthase, responsible for synthesis of the autoinducer molecule, *N‐*(3‐oxohexanoyl)‐L‐homoserine lactone (N‐Acyl homoserine lactone, AHL); the latter codes for the transcriptional activator of the bioluminescence operon. When the autoinducer molecule binds to LuxR, they activate transcription of the entire *lux* operon, including their own genes [21]. As schematically presented in Figure 1, we have designed a circuit in which the *yqjF* or the *azoR* gene promoters are integrated with these quorum‐sensing elements; this design is derived from a previously described *A. fischeri*‐based two‐plasmid synthetic QS circuit [22]. Several host *E. coli* strains and several variants of the basic circuit designs were tested in several combinations, yielding new reporter strains that exhibited a significantly improved DNT detection sensitivity, with a detection threshold over 300 fold lower than that of the original bioreporter.

## 2 MATERIALS AND METHODS

### 2.1 Chemicals

2,4‐Dinitrotoluene (DNT, Cat.101397) and all other chemicals were purchased from Sigma‐Aldrich and were of the highest analytical grade. DNT was dissolved in ethanol at a concentration of 27 g/L, and kept at room temperature. Restriction enzymes and other DNA modification enzymes were purchased from New England Biolabs (Ipswich, MA, USA) and DNA purification kits from Qiagen (Germantown, MD, USA). *N‐*(3‐Oxododecanoyl)‐homoserine lactone (AHL‐C6) and *N‐*(β‐Ketocaproyl)–homoserine lactone (AHL‐C12) were purchased from Sigma‐Aldrich.

### 2.2 Bacterial strains and plasmid construction

Bacterial host strains and plasmids constructed and/or used in this study are summarized in Table 1; primers employed for the construction of these plasmids and for modifying the host strains are listed in Table S1. All cloning products were verified by sequencing. Plasmids and bacteria were maintained by growing the bacteria in the presence of relevant antibiotics: ampicillin (100 μg/mL), kanamycin (50 μg/mL), or chloramphenicol (30 μg/mL).

**TABLE 1:** Bacterial strains and plasmids used in this study

	**Bacterial host strain**	**Description**	**Reference**
	*E. coli* DH5α	*fhuA2 Δ(argF‐lacZ)U169 phoA glnV44 Φ80 Δ(lacZ)M15 gyrA96 recA1 relA1 endA1 thi‐1 hsdR17*	New England Biolabs
	*E. coli* MG1655	F^–^ *1^–^ ilvG‐rfb‐50, rph‐1*	[29]
	*E. coli* RFM443	*strR, galK2, lacD74*	[26]
	*E.coli* BW25113 ΔpykF	Chromosomal deletion of *pykF* (Kan^R^)	[30]
	*E.coli* BW25113 ΔygdD‐ΔeutE	Chromosomal deletion of *ygdD* and *eutE* (Kan^R^)	[11]
**#**	**Plasmids (** **Figure S1**)	**Description**	**Reference**
A	pluxRI2	*luxR* and *luxI* under lac/ara promoters (chloramphenicol resistance)	[22]
B	pluxRI2‐azoR	*luxR* and *luxI* under azoR promoter (chloramphenicol resistance)	This work
C	pluxRI2‐azoR‐ygjF(C55)	*luxR* under azoR and *luxI* under yqjF (C55) promoters (“AND Gate”) (chloramphenicol resistance)	This work
D	pluxRI2‐2PG3‐65	*luxR* and *luxI* under 2PG3‐65 promoter (chloramphenicol resistance)	This work
E	pluxRI2‐C55/2PG3‐65/71‐yhaJ(G2)	*luxR* and *luxI* under C55/2PG3‐65/71 promoters; yhaJ(G2) (chloramphenicol resistance)	This work
F	luxI‐lacZ‐CcdB3	lacZ‐CcdB3 under luxI promoter (kanamycin resistance)	[22]
G	luxI‐luxPl	*P. luminescens luxCDABE* under luxI promoter (kanamycin resistance)	This work
H	luxI‐luxPl‐Amp	*P. luminescens luxCDABE* under luxI promoter (ampicillin resistance)	This work
I	luxI‐luxAf	*A. fischeri luxCDABEG* under luxI promoter (ampicillin resistance)	This work
J	luxI‐luxI‐luxAf	*A. fischeri luxCDABEG* and luxI under luxI promoter (ampicillin resistance)	This work
K	luxI‐luxR‐luxAf	*A. fischeri luxCDABEG* and luxR under luxI promoter (ampicillin resistance)	This work
L	luxI‐luxRI2‐luxAf	*A. fischeri luxCDABEG*, luxI and luxR under luxI promoter (ampicillin resistance)	This work
M	pBR‐C55‐luxAf	*A. fischeri luxCDABEG* under yqjF (C55) promoter (ampicillin resistance)	[14]
N	pACYC‐yhaJ(G2)	yhaJ gene and promoter following 2 rounds of mutagenesis (chloramphenicol resistance)	[11]

The basic two‐plasmid QS system was kindly donated by Prof. Frances Arnold (California Institute of Technology, Pasadena, CA, USA [22]). Plasmid p*luxRI2* harbors the *luxR* and *luxI* genes downstream to the *lac/ara* promoter (Table 1, row A); the LuxR‐AHL complex induces the *luxI* promoter of the second plasmid (luxI‐lux) (Figure 1). As described in Sections 2.3 and 2.4 below, several modifications were introduced in the course of this work to both QS plasmids, in order to shift their sensing target to DNT and enhance their sensitivity, generating the plasmid variants listed in Table 1.

### 2.3 Construction of pluxRI2‐based plasmids

Four different variants (Table 1, rows B–E) based on the original pluxRI2 plasmid (Table 1, row A) were constructed, in all of which the *lac/ara* promoter was replaced by DNT‐responsive promoters. In plasmid pluxRI2‐azoR (Table 1, row B), the *lac/ara* was replaced by *azoR*, a native *E. coli* promoter previously found to be DNT‐inducible [23]. This was performed by digesting the pluxRI2 plasmid with restriction enzymes *BamHI* and *NotI*, thus removing the *lac/ara* promoter, and replacing it with the *azoR* promoter, obtained with PCR amplification from *E. coli* WT MG1655 genomic DNA (primers PazoR_F_XhoI and PazoR_R_EcoRI, Table S1).

In plasmid pluxRI2‐azoR‐yqjF(C55) (Table 1, row C), the *luxR* gene remained downstream to the *azoR* promoter, but *luxI* was placed under the C55 version of the *yqjF* gene promoter [9, 11]. This “AND gate” was constructed in an attempt to reduce the system's background luminescence. Construction of this plasmid was performed in two consecutive steps. First, to place the azoR‐luxR segment in opposite directionality to the *luxI* gene, this segment was PCR‐amplified using primers 26_azoR_Bam_F and 27_luxR_Sal_R (Table S1). The resulting 0.9 kb fragment was digested with SalI and BamHI restriction enzymes and ligated into pluxRI2‐azoR (Table 1, row B), predigested with the same enzymes. To allow further modifications, BamHI and SmaI restriction sites were introduced between *luxI* and the *azoR* promoter. Then, the *yqjF* (C55) promoter was PCR‐amplified from pBR‐C55‐lux [11] with primers 21_C55_Bam_R and 13_kpn_F (Table S1), yielding a 260 bp product. This product, as well as the plasmid from the previous step, were digested with *BamHI* and *SmaI* restriction enzymes, and then ligated to form the “AND gate” pluxRI2‐azoR‐yqjF(C55) plasmid (Table 1, row C).

In another attempt to reduce background luminescence and improve the sensitivity of the bioreporter, the *lac/ara* promoter in the pluxRI2 plasmid (Table 1, row A) was replaced by the 2PG3‐65 promoter. This promoter is the result of several rounds of error‐prone PCR coupled to DNA shuffling, previously performed on a DNA segment containing a fusion between the C55 promoter and a mutated version of the *azoR* promoter [12]. The 2PG3‐65 promoter, which was shown to exhibit a lower background and a higher sensitivity than the C55 promoter, was PCR‐amplified (primers 162_C55_Sal_F and 163_RBS_R, Table S1) from pBR‐2PG3‐65‐lux [12]. It was then ligated into plasmid pluxRI2‐azoR (Table 1B), predigested with SalI and KpnI, using the Gibson assembly technique (New England Biolabs). The new construct was denoted pluxRI2‐2PG3‐65 (Table 1, row D).

An additional attempt at improving the bioreporter's performance was conducted by introducing into the same vector an enhanced version of the *yhaJ* gene, previously found to act as an activator of DNT‐related genes [12, 24], enhanced by two rounds of directed evolution [13]. Cloning was performed by PCR amplification of *yhaJ* (G2) using primers 186_yhaJ_SphI_F and 187_yhaJ_SalI_R (Table S1). The resulting 1.2 kb‐PCR fragment was cloned into pluxRI2‐2PG3‐65, predigested by SalI, using the Gibson assembly technique. Two additional DNT‐responsive promoters, C55 and 2PG3‐71 [12], were also cloned upstream to the yhaJ (G2) segment. The new construct was denoted pluxRI2‐2PG3‐65‐yhaJ(G2) (Table 1 row E).

### 2.4 Construction of pluxI‐lux‐based plasmids

Plasmid luxI‐luxPl (Table 1 row G) was constructed by replacing the *lacZ* reporter gene in the original lacZ‐CcdB3 plasmid [22] with the *luxCDABE* gene cassette of *Photorhabdus luminescens*. The lacZ‐CcdB3 sequence was excised using restriction enzymes *StuI* and *KpnI*, and replaced with *luxCDABE*, amplified by PCR from plasmid *pBR2TTS* [10] with primers luxCDABE_F_StuI and luxCDABE_R_KpnI (Table S1), and digested with the same enzymes.

To allow further signal enhancements by introducing specific gene mutations into the host genome [11], the kanamycin resistance cassette of the original plasmid was replaced by an ampicillin resistance gene. The *ampR* gene was PCR‐amplified from plasmid pBR‐C55‐lux [11] using primers 115_Amp_R and 116_Amp_F (Table S1) and the luxI‐luxPl fragment was PCR‐amplified from its target plasmid (Table 1, row G) using primers 117_pluxI_F and 118_pluxI_R (Table S1). The two PCR products were assembled using the Gibson assembly technique; the new plasmid was listed as luxI‐luxPl‐Amp (Table 1 row H).

The *P. luminescens lux* cassette in plasmid pluxI‐luxPl‐Amp was replaced with the *luxCDABEG* genes of the marine bacterium *A. fischeri* (luxAf), previously shown to yield stronger luminescence at temperatures below 30°C [14], to generate plasmid luxI‐luxAf (Table 1, row I). To this end, the *luxAf* cassette from plasmid pBR‐C55‐luxAf (Table 1, row M) was PCR‐amplified using primers 119_luxAf_F and 120_luxAf_R (Table S1). The 6 kb‐purified PCR product was cloned into the luxI‐luxPl‐Amp plasmid, predigested with NotI and kpnI, using the Gibson assembly technique.

A series of additional constructs were designed to better mimic the natural quorum‐sensing mechanism present in bacteria such as *A. fischeri*. This was performed by fusing the *luxI* and *luxR* genes to the *luxI* promoter in various combinations. To this end, luxI‐luxAf was digested with *SfoI* and *KpnI* restriction enzymes and the various DNA segments were PCR‐amplified and cloned into the digested vector using the Gibson assembly cloning technique as follows:

(i) luxI‐luxI‐luxAf (Table 1, row J), containing the *luxI* gene between the *luxI* promoter and the *luxAf* cassette, was constructed by amplifying the *luxI* gene from plasmid pUCD615 [25] with primers 122_luxI_R and 136_luxR_F (Table S1).
(ii) luxI‐luxR‐luxAf (Table 1, row K), containing the *luxR* gene under the control of the *luxI* promoter, but in opposite direction to the *luxAf* cassette, was constructed by amplifying *luxR* from plasmid luxI‐luxRI2‐Af with primers 147_luxR_R and 161_luxI_Kpn_R (Table S1), cloning it into plasmid luxI‐luxAf, in the opposite direction to the *luxAf* cassette.
(iii) luxI‐luxRI2‐Af (Table 1, row L), containing both the *luxI* and *luxR* genes between the *luxI* promoter and the *luxAf* cassette, was constructed in three steps as follows:
a. *luxI* and *pluxI* (gene and promoter) were PCR‐amplified from plasmids luxI‐luxI‐Af in two steps, first using primers 122_luxI_R and 148_luxI_R, and then using the amplicon as a target for a second PCR step with primers 122_luxI_R and 149_luxI_R2 (Table S1).
b. The *luxR* gene was amplified from plasmid pluxRI2‐azoR with primers 147_luxR_R and 150_luxR_F (Table S1).
Gibson assembly cloning was conducted using the SfoI/KpnI‐digested vector and PCR products from Steps I and II.

Two additional plasmids listed in Table 1 are pBR‐C55‐luxAf (row M) and pACYC‐yhaJ(G2) (row N). The former contains the *A. fischeri luxCDABEG* cassette driven by the *yqjF* (version C55) gene promoter, and the latter expresses the YhaJ protein, the transcriptional regulator of *yqjF* [11].

### 2.5 Measuring the bioreporters’ response to DNT

The plasmids constructed as outlined in the previous section and listed in Table 1 were chemically transformed into *E. coli* hosts to generate a 12‐member panel of QS bioreporter strains (QBS, Table 2). To assay their bioluminescent response to DNT, all strains were grown overnight in lysogeny broth (LB), supplemented with either ampicillin, chloramphenicol, or kanamycin (100, 30, and 50 mg/L, respectively), at 37°C with shaking (200 rpm). The culture was then diluted 100‐fold in fresh LB without antibiotics, and further incubated under the same conditions until reaching an optical density at 600 nm (OD_600_) of ca. 0.3. In experiments where different bacterial cell densities were required, incubation continued until reaching the indicated optical density. Duplicate bacterial aliquots (50 μL) were mixed with 50 μL of serial dilutions of DNT in 4% (v/v) ethanol (2% final ethanol concentration) in opaque white 96‐well microtiter plates (Greiner Bio‐One). Bioluminescence was measured using a microplate reader (Infinite 200PRO, Tecan, Switzerland or VICTOR^2^, Wallac, Turku, Finland) every 15 min at ambient temperature. Each measurement was accompanied by vigorous shaking of the plate. All experiments were repeated at least three times.

**TABLE 2:** Quorum–sensing bioreporters (QSB) used in this study

**Quorum sensing bioreporter (QSB)**	**Plasmid 1 (**pluxRI2 derivative)^a^	**Plasmid 2 (**luxI‐lux derivative)^a^	**Bacterial host strain**
**1**	pluxRI2‐azoR (B)	luxI‐luxPl (G)	*E. coli* RFM443
**2**	pluxRI2‐azoR (B)	luxI‐luxPl‐Amp (H)	*E.coli* BW25113 ΔpykF
**3**	pluxRI2‐azoR‐ygjF(C55) (C)	luxI‐luxPl‐Amp (H)	*E.coli* BW25113 ΔpykF
**4**	pluxRI2‐azoR‐ygjF(C55) (C)	luxI‐luxAf (I)	*E.coli* BW25113 ΔpykF
**5**	pluxRI2‐azoR‐ygjF(C55) (C)	luxI‐luxRI2‐luxAf (L)	*E.coli* BW25113 ΔpykF
**6**	pluxRI2‐azoR‐ygjF(C55) (C)	luxI‐luxI‐luxAf (J)	*E.coli* BW25113 ΔpykF
**7**	pluxRI2‐2PG3‐65 (D)	luxI‐luxAf (I)	*E.coli* BW25113 ΔpykF
**8**	pluxRI2‐2PG3‐65‐yhaJ(G2) (E)	luxI‐luxAf (I)	*E.coli* BW25113 ΔygdD‐ΔeutE
**9**	pluxRI2‐2PG3‐71‐yhaJ(G2) (E)	luxI‐luxAf (I)	*E.coli* BW25113 ΔygdD‐ΔeutE
**10**	pluxRI2‐C55‐yhaJ(G2) (E)	luxI‐luxAf (I)	*E.coli* BW25113 ΔygdD‐ΔeutE
**11**	pACYC‐yhaJ(G2) (N)	pBR‐C55‐luxAf (M)	*E.coli* BW25113 ΔygdD‐ΔeutE
**12**	pluxRI2 (A)	–	*E. coli* DH5α

^a^Letter in parentheses refers to Table 1 row number.

### 2.6 Response of QS elements to acyl‐homoserine lactone

Two types of synthetic acyl‐homoserine lactones (AHL) were tested: *N‐*(3‐Oxododecanoyl)‐homoserine lactone (AHL‐C6) and *N*‐(β‐Ketocaproyl)–homoserine lactone (AHL‐C12). Both compounds were dissolved in 100% ethanol at a concentration of 100 μM. Further dilutions were made with H_2_O. Aliquots of AHL were added to the QSB10 and QSB11, grown as described in the previous section, and the activation of the *lux* cassette in response to the supplemented AHL was similarly monitored.

To evaluate the effect of the AHL produced by the two‐plasmid QS system, a strain (QSB12) harboring only the original pluxRI2 plasmid, in which both *luxR* and *luxI* are under *lac*/*ara* promoters (Table 1, row A), was employed. For AHL production, this strain was grown overnight (200 rpm) at 37°C in LB containing 1 mM IPTG and 0.5% arabinose. The culture was then centrifuged (3200 × *g*, 10 min, 4°C), the supernatant was removed, and its effect on strains QSB10 and QSB11 was tested as described before. Medium similarly removed from an overnight culture with neither IPTG nor arabinose was used as a control.

### 2.7 Bioreporter responses to DNT on a solid matrix

The response of bacterial bioreporters to DNT on a solid surface was measured by immobilizing the bacteria in 1.5‐mm 2% (v/v) alginate/1% (v/v) polyacrylic acid beads, as previously described [14]. Bacterial concentration in the beads, kept at 4°C, was ca. 1.5 × 10^5^ cells per bead. The solid matrix was LB‐agar (800 μL per well) in a 24‐well microtiter plate (Greiner, Bio‐One), supplemented with 10 μL of 100% ethanol containing different amounts of DNT. To allow ethanol evaporation, the 24‐well plates were left open in a chemical hood for 1 h. Prior to the experiment, the encapsulated bacteria were removed from refrigeration and incubated in LB for 2 h at 30°C with shaking (200 rpm). The beads were then drained of medium, and placed in a single layer over the LB‐agar surface of the microtiter plate wells (20–30 beads per well). Bioluminescence was measured every 15 min in a microplate reader (Infinite 200 PRO, Tecan) at room temperature.

### 2.8 Calculations

Luminescence data are presented in the plate reader's arbitrary relative light units (RLU); they are also displayed as the difference in the intensity of the signal in the presence and absence of the inducer (ΔRLU) or as the response ratio, the luminescence in the presence of the inducer divided by that in its absence. The DNT detection sensitivity of each strain was determined by calculating an EC_200_ value, the DNT concentration at which luminescence intensity is two‐fold higher than that of the uninduced control [26, 27].

## 3 RESULTS

### 3.1 Performance of the DNT sensor strains

As mentioned in Section 2.2 above, each of the sensor strains constructed in the course of the present study harbored two compatible plasmids (Figure 1). Different combinations of the two plasmids were introduced into four *E. coli* host strains to generate eleven bioreporters (QSB1‐QSB11, Table 2), which were tested for their response to DNT. In the first six of these reporters, QSB1‐QSB6, the *azoR* gene promoter serves as the DNT‐inducible sensing element, either on its own (QSB1,2), or in combination with the C55 variant of the *yqjF* gene promoter (QSB3‐6). The *P. luminescens luxCDABE* gene cassette is the bioluminescent reporter in QSB1‐3, and *A. fischeri*’s *luxCDABEG* in the rest of the strains (QS4‐11). Except for QSB1, all reporter strains carry either a single mutation in the *pykF* gene (QSB2‐7) or a double *ygdD‐eutE* mutation (QSB8‐11), previosly shown [11] to enhance the responses to DNT.

**FIGURE 2:**
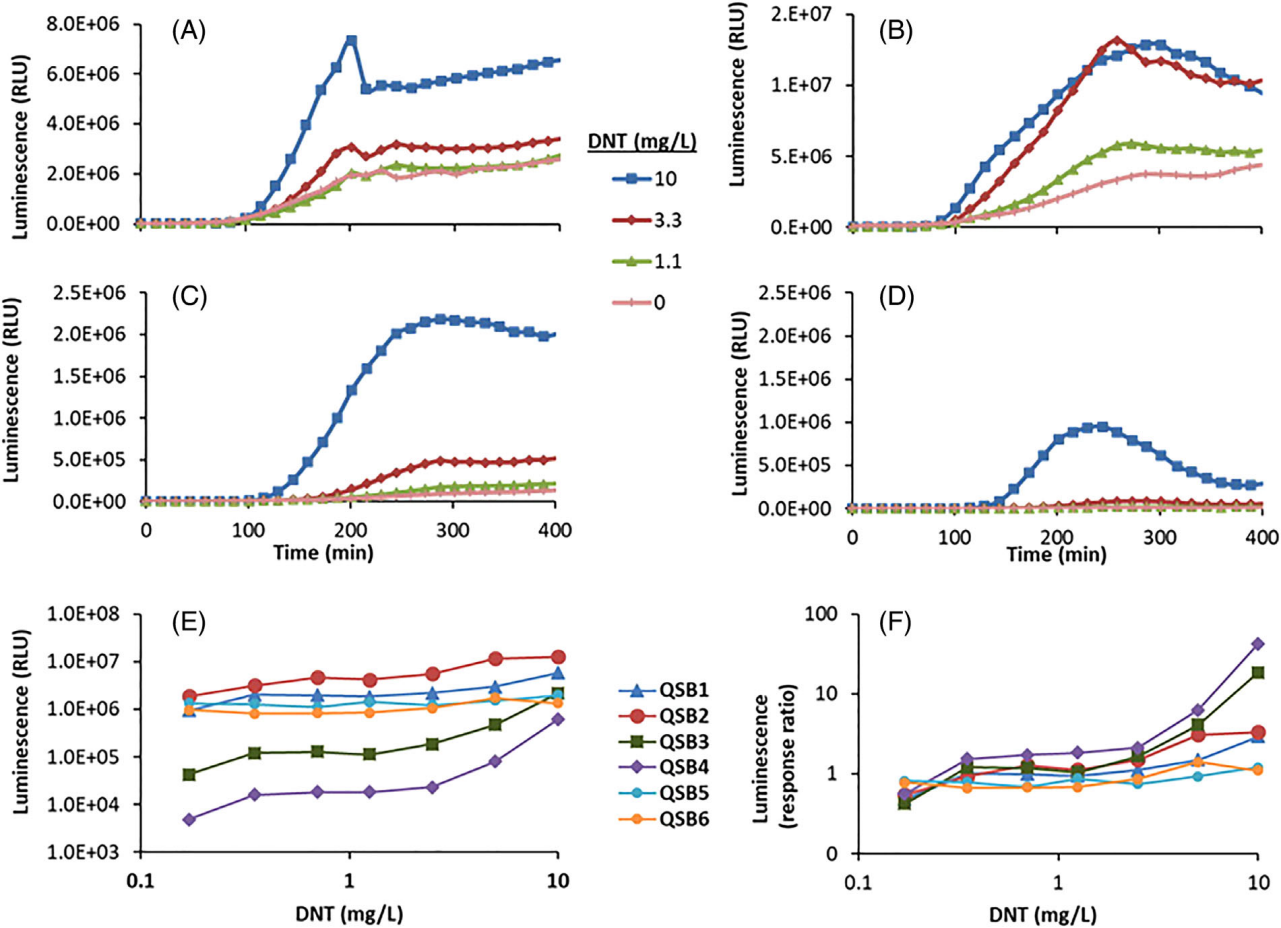
Typical signal development of the QS‐system, (A) QSB1, (B) QSB2, (C) QSB3, and (D) QSB4. DNT‐induced luminescence in sensor strains QSB1‐QSB6: maximal average signal intensities (E) and maximal average response ratios (F) over a 400 min exposure period are presented as a function of DNT concentration. Luminescence values in panels A–D are in the plate reader's arbitrary relative light units (RLU). All experiments were repeated at least three times, with the standard deviation not exceeding 15% in all cases. The time‐dependent curves in Panels A–D are representative examples. In panels E and F, standard deviations are not marked on the curves for the sake of clarity

Figure 2 displays luminescent signal development in the presence of DNT as a function of time in reporter strains QSB1‐QSB4 (Figure 2A–D), as well as the dependency of these reactions on DNT concentration (Figure 2E and F). Highest signal intensities were displayed by QSB2, and the lowest by QSB4. These differences were paralleled by the intensity of the uninduced control, and were thus reflected in the calculated response ratios (Figure 2F). As shown in Figure 2E, reporter strains QSB5 and QSB6, though strongly luminescent, showed no significant response to DNT. Strain QSB4, which displayed the highest response ratio in almost the entire DNT concentration range (Figure 2F), was also characterized by the lowest luminescence intensities (Figure 2E), a fact that detracted from its value as a potential DNT sensor for future field applications.

**FIGURE 3:**
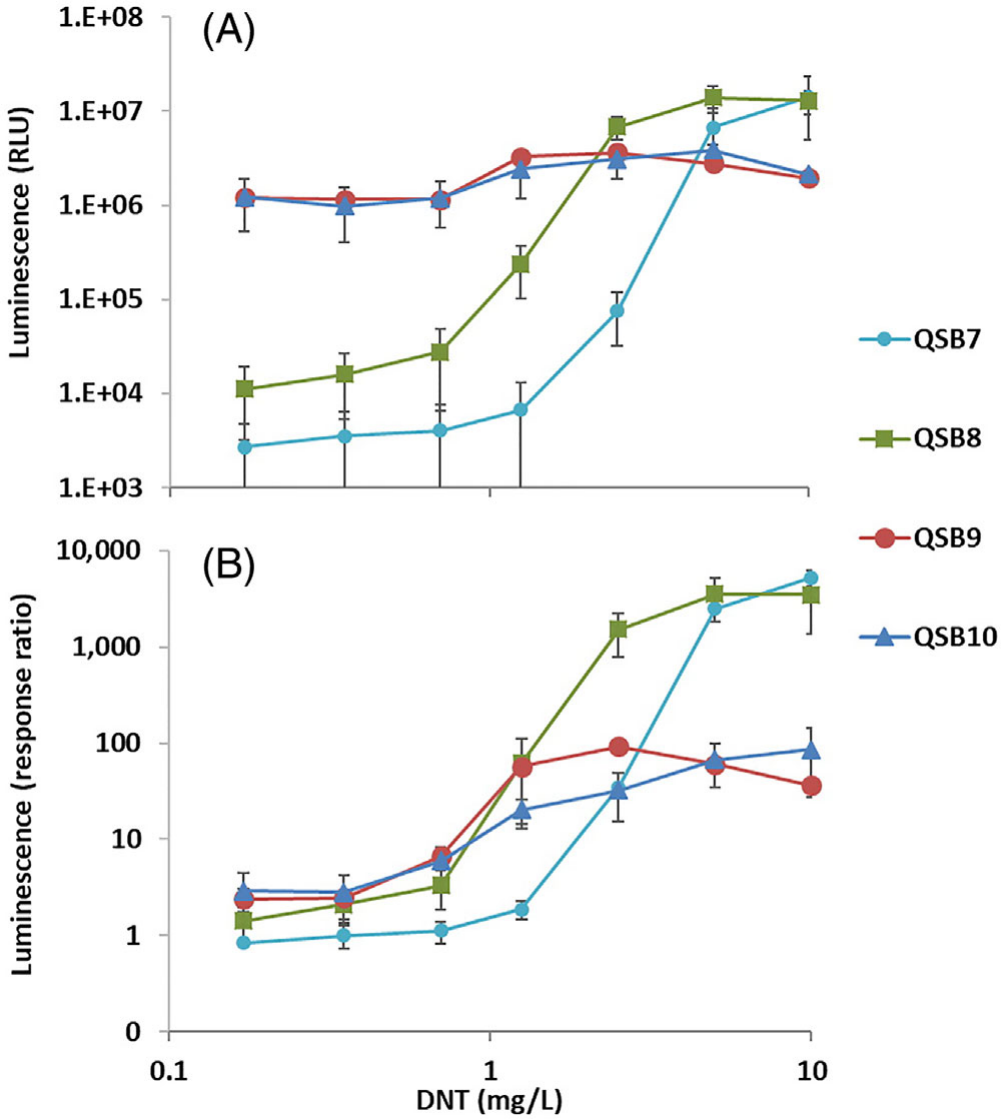
DNT‐induced luminescence in sensor strains QSB7‐QSB10. Maximal average signal intensities (A) and maximal average response ratios (B) over a 400 min exposure period are presented as a function of DNT concentration. Luminescence values in panel A are in the plate reader's arbitrary relative light units (RLU). All experiments were repeated at least three times. Error bars denote the standard deviation

In sensors QSB7 to QSB10, the *azoR/yqjF* sensing element combinations have been replaced by alternative sensing elements (Table 2); in addition, *ygdD* and *eutE* mutations have been introduced to the host strain in QSB8‐10, replacing the *pykF* mutations. The responses of these four bioreporters to DNT are presented in Figure 3, in terms of both signal intensity (Figure 3A) and the fold‐induction (response ratio) over the uninduced control (Figure 3B).

**FIGURE 4:**
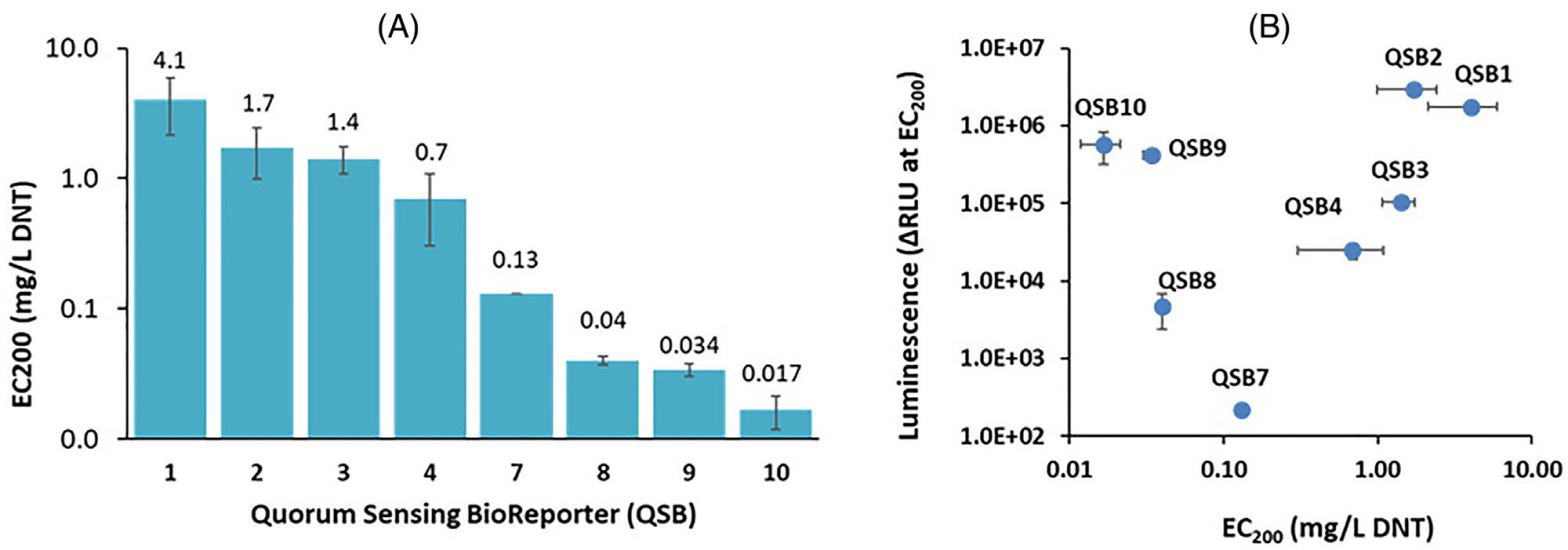
DNT detection sensitivity and signal intensity in the QS‐based bioreporters. (A) Detection sensitivity, presented as the EC_200_ value (DNT concentration at which luminescence is twice that of the control). (B) Signal intensity (ΔRLU) at the EC_200_ point, as a function of EC_200_. All experiments were repeated at least three times; error bars denote the standard deviation

The detection sensitivity of all bioreporters listed above is displayed in Figure 4A as the EC_200_ value – the DNT concentration at which the intensity of the luminescent response is twice that of the control (response ratio = 2). As may be observed, the introduction of the *pykF* mutation in QSB2‐QSB4 has led to an increase in detection sensitivity of ca 3‐fold to 8‐fold compared to the starting strain, QSB1. Replacing the *a* *zoR/yqjF* combination with the 2PG3‐65 promoter in QSB7 has led to a further decrease in the EC_200_ value, from 0.7 mg/L in QSB6 to 0.13 mg/L. The next step in sensitivity enhancement was caused by the shift to the *ygdD/eutE* double mutation in the host: EC_200_ values of 0.04 mg/L, 0.034 mg/L and 0.017 mg/L were calculated for reporter strains QSB8, QSB9, and QSB10, respectively. The latter value is over 350‐fold lower than the initial QSB1 sensor.

In Figure 4B, the luminescence intensity of the different sensor strains in response to DNT concentrations equal to the EC_200_ value, is presented a function of the EC_200_. Variants QSB9 and QSB10 stand out by the combination of a high sensitivity and a strong luminescence intensity.

### 3.2 Effect of AHLs

**FIGURE 5:**
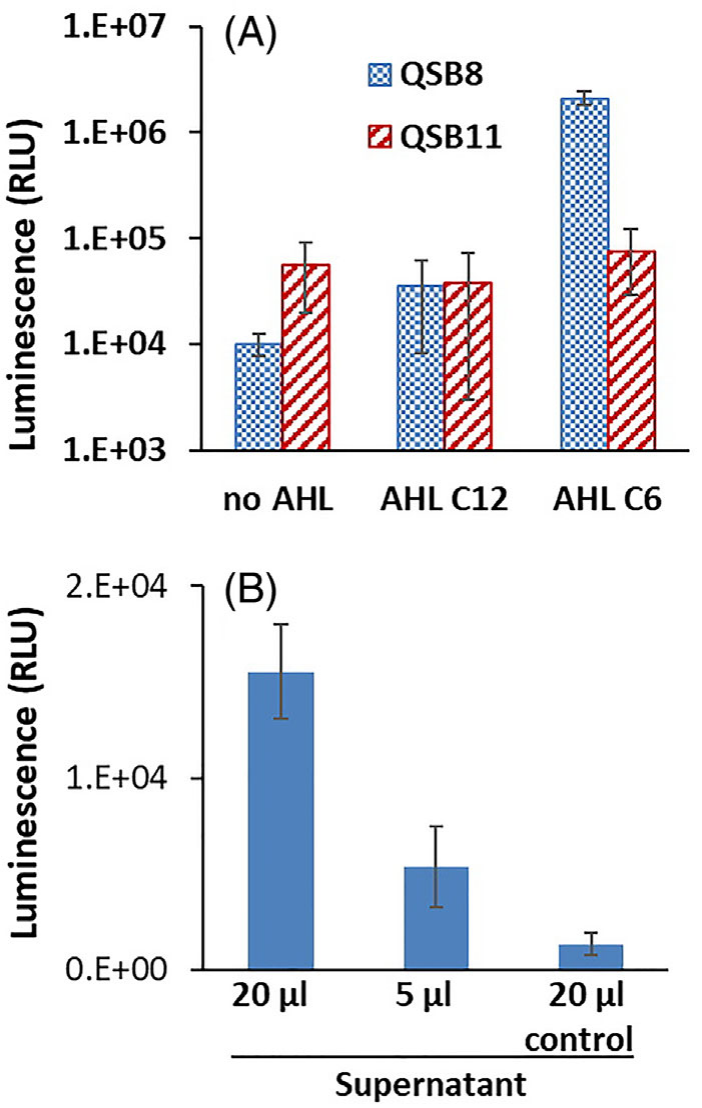
(A) Effect of acyl‐homoserine lactones AHL‐C6 and AHL‐C12 (100 nM) on the luminescence of a QS‐based sensor (QSB8) and a non‐QS sensor (QSB11). (B) Effect on QSB8 luminescence of a supernatant (5 and 20 μL) of an overnight *E. coli* culture of strain QSB12, harboring the pluxRI2 plasmid, induced by 1 mM IPTG and 0.5% Arabinose. Luminescence values are in the plate reader's arbitrary relative light units (RLU). Error bars denote the standard deviation

AHLs are the small‐molecule autoinducers of the QS system. *A. fischeri*’s AHL is *N‐*(3‐Oxododecanoyl)‐homoserine lactone (AHL‐C6), whereas *N‐*(β‐Ketocaproyl)‐homoserine lactone (AHL‐C12) serves a similar purpose in *Pseudomonas aeruginosa* [28]. The effect of the two compounds at a concentration of 100 nM on strain QSB8 was investigated, in comparison to the control strain QSB11 (non‐QS strain). As expected, the *A. fischeri*‐based QS system (QSB8) was much more sensitive to AHL‐C6 than to AHL‐C12 (Figure 5A): the increase in luminescence to AHL C12 and C6 was 10‐fold and over 100‐fold higher, respectively, than the AHL‐free control. No such response was observed in the QSB11 control system.

Further confirmation of the AHL‐dependency of the system was conducted using the supernatant of an IPTG/arabinose‐induced culture of strain QSB12, harboring only the original pluxRI2 plasmid, in which both *luxR* and *luxI* are under the lac/ara promoters. As shown in Figure 5B, addition of 20  and 5 μL of the supernatant resulted in a strong response of QSB8; in the presence of 20 μL of the supernatant, response intensity was over 30‐fold higher than that of the control; there was no response of the control non‐QS strain QSB11 (data not shown).

### 3.3 Bioreporter responses to DNT on a solid matrix

**FIGURE 6:**
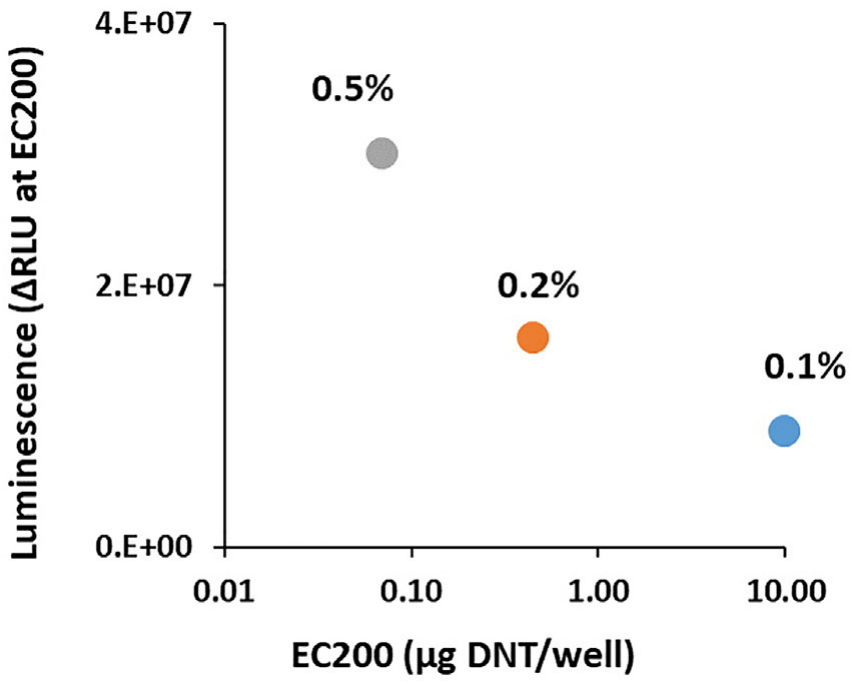
Detection of DNT on a solid medium: effect of bacterial concentration. Sensor strain QSB10 was encapsulated in 1.5 mm alginate beads, and placed on the surface of LB agar in 24‐well microtiter plates, containing different DNT concentrations. Bacterial densities are depicted as percent bacterial pellet weight in alginate (0.1% ∼ 1.5 × 10^5^ cells per bead). Signal intensity (ΔRLU) at the DNT concentration equal to EC_200_ is plotted against the EC_200_ values. Presented data are an average of duplicates

A possible future implementation of buried explosives’ detection by bioreporter bacteria is likely to involve the encapsulation of the bacteria in a permeable polymeric matrix [4, 14]. To preliminarily demonstrate the potential applicability of the QS‐based sensor system in such a scheme, strain QSB10 was encapsulated in 1.5 mm alginate/polyacrylate beads as described above in Material and Methods, and exposed to DNT on a solid LB agar surface. Different bacterial densities were tested: 0.1%, 0.2% and 0.5% of bacterial pellet in the alginate solution. The lowest concentration, 0.1% (ca. 1.5 × 10^5^ cells/bead) was previously shown [14] to be optimal for the non‐QS system (QSB11). As can be seen in Figure 6, elevating the bacterial density in the beads resulted in both a higher signal intensity and a lower detection threshold. When the same experiment was conducted using the non‐QS control strain QSB11, signal intensity actually declined at the higher cell densities, and sensitivity remained unaffected (data not shown).

## 4 DISCUSSION

In the study reported herein, we have attempted to deviate from the simplistic promoter‐reporter fusion characteristic of most bacterial sensor strains; this was achieved by introducing the *luxI* and the *luxR* genes from the *lux* operon of the luminescent marine bacterium *Alliivibrio fischeri*. These two genes, the main players in regulating the quorum‐sensing mechanism of *A. fischeri*, were employed in a two‐plasmid system: in one of them, a 2,4‐dinitrotoluene (DNT)‐sensitive promoter induced both *luxI* and *luxR*, and in the other the *lux* reporter cassette was driven by the *luxI* promoter, activated by the AHL‐LuxR pair. The inducer of the system, DNT, is an excellent signature chemical for the presence of buried landmines, and the sensors constructed in the course of this study may serve as potential sensor strains in a future standoff detection scheme.

As demonstrated in the results presented above, the QS‐based sensor design functioned as expected, and a clear dose‐dependent response was observed for at least a part of the tested DNT concentration range. To confirm the involvement of quorum‐sensing elements in this detection system, we have demonstrated its response to an externally supplied *A. fischeri* AHL‐C6 autoinducer (Figure 5A); furthermore, it was also strongly induced by a culture supernatant containing a naturally excreted autoinducer (Figure 5B).

Major improvements in the sensor's performance were achieved by manipulating the non‐QS elements of the circuit: the DNT‐inducible promoter driving *luxI* and *luxR* expression, the bioluminescence gene cassette driven by the *luxI* promoter, and the host strain's genome. As shown in Figure 4, this process has led to the construction of strain QSB10, characterized by a signal intensity that was on the one hand three‐fold lower than strain QSB1 (at the threshold DNT concentration), but on the other hand displayed a detection threshold that was over 300 fold lower.

Earlier optimization studies of the alginate encapsulation of *E. coli*‐based DNT sensor strains [14] have indicated as optimal a bacterial density of ca 1.5 × 10^5^ cells per a 1.5 mm bead. Higher cell densities were detrimental to the activity, possibly due to oxygen limitation. In the present case, a higher cell density appeared to be beneficial in terms of both signal intensity and detection sensitivity. It is likely that this effect was due to the accumulation of AHL molecules in the bead matrix, a hypothesis that was not tested in the present study. This phenomenon may allow to enhance the performance of encapsulated sensor cells in future field applications by using them at concentrations beyond the constraints imposed by earlier studies.

Using different *E. coli*‐based bioreporters, we have previously demonstrated the detection of 0.33 μg of DNT in 0.8 mL of solid LB agar [12], as well as the successful identification of the presence of a buried antipersonnel landmine [14]. Concentrations of TNT and DNT above buried explosive devices and in munitions‐polluted soils vary greatly, depending upon mine type, soil characteristics, and environmental conditions such as humidity and temperatures (1). While in some cases DNT concentrations as high as 800 μg/Kg soil were measured, in others the reported levels were as low as 1 μg/Kg soil (6–8). The bacterial bioreporters described in the present communication, displaying a detection limit around 20 μg/L, should adequately detect trace explosives at the mid to high segment of this concentration range (moist soils, plastic mines) but will need additional sensitivity enhancement to function at the lower concentration range (dry soils, metal mines).

## CONFLICT OF INTEREST

The authors declare no conflict of interest.

## Supporting information

Supporting Information

Supporting Information

## Data Availability

The data that support the findings of this study are available from the corresponding author upon reasonable request.
