# Supplementary material for: Introduction of quorum sensing elements into bacterial bioreporter circuits enhances explosives’ detection capabilities
Source: Eng Life Sci. 2022 Mar 2;22(3-4):308–18. doi: 10.1002/elsc.202100134 (PMC8961053; doi:10.1002/elsc.202100134)
Supplement: Supplementary file 1 — Supporting Information [file ELSC-22-308-s002.docx]

Figure S1. Schematic structures of the plasmids constructed and employed in the course of the present study.

1. pLuxRI2-based plasmids

**B**

***pLuxRI2-pazoR***

***CmR***

***luxI***

***luxR***

**_P_azoR**

**ColE1**

***pLuxRI2-pazoR-C55***

***CmR***

***luxI***

***luxR***

**_P_azoR**

**ColE1**


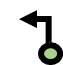

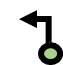


**_P_C55**

**+**

**A**

***pLuxRI2***

***CmR***

***luxI***

***luxR***

**_P_lac/ara**

**ColE1**

**C**

**D**

***pLuxRI2-2PG3-65***

***CmR***

***luxI***

***luxR***

**_P_2PG3-65**

**ColE1**

**E**

***pLuxRI2-2PG3-65-yhaJ(G2)***

***CmR***

***luxI***

***luxR***

**_p_C55/_P_2PG3-65/71**

**ColE1**

***yhaJ(G2)***

1. pLuxI-luxPl plasmids

**_p_luxI-luxRI2**

**_p_luxI-luxR**

**_p_luxI-luxI**

**G**

***pLuxI-luxPl***

**_p_luxI**

***KanR***

***luxCDABE (P. luminescens)***

p15A

**H**

***pLuxI-luxPl-Amp***

**_p_luxI**

***AmpR***

***luxCDABE (P. luminescens)***

p15A

**I**

***pLuxI-luxAf***

**_p_luxI**

***AmpR***

***luxCDABEG (A. fischeri)***

p15A

***luxI***


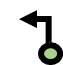

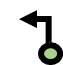


***luxR***


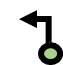

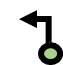


***luxR***

***luxI***


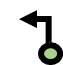


**J**

**K**

**L**

**F**

***pLuxI-lacZ-CcdB3***

**_p_luxI**

***KanR***

***lacZ-CcdB3***

p15A
