# Supplementary material for: Introduction of quorum sensing elements into bacterial bioreporter circuits enhances explosives’ detection capabilities
Source: Eng Life Sci. 2022 Mar 2;22(3-4):308–18. doi: 10.1002/elsc.202100134 (PMC8961053; doi:10.1002/elsc.202100134)
Supplement: Supplementary file 2 — Supporting Information [file ELSC-22-308-s001.docx]

Table S1. Primers employed in the construction of the sensor plasmids and bioreporter strains described in this article

|  | Primer name | Sequence 5’🡪3’ |
| --- | --- | --- |
|  | **PazoR_F_XhoI** | CCATGACTCGAGCAGTCTGTCACGCAGCATC |
|  | **PazoR_R_EcoRI** | CCATGAGAATTCGCCAGGATGCTGGATTTAAG |
|  | **luxCDABE_F_StuI** | CGTCAGAGGCCTTCTGTCATGCCAAACTTTGC |
|  | **luxCDABE_R_KpnI** | CCAGTGGTACCTCAGGAGGGGCAAATATGAC |
|  | **26_azoR_Bam_F** | tttttGGATCCTCGCCCTATAGTGAGTCGTATTACCCGGGTCG AGC AGT CTG TCA CGC A |
|  | **27_luxR_Sal_R** | tttttGTCGACCGTACTTAATTTTTAAAGTATGGGCAATC |
|  | **13_kpn_F** | GATCGGGATCCCCGGGTACC |
|  | **21_C55_Bam_R** | TTTTTGGATCCGCCACTCAGGCTGCTGATTG |
|  | **115_Amp_R** | AGTTGTTACTAGTCTACGGGGTCTGACGCTC |
|  | **116_Amp_F** | GGCTTTCTTGCCCTCGTGATACGCCTATTTTTATAGG |
|  | **117_pluxI_F** | GGCGTATCACGAGGGCAAGAAAGCCATCCAGTTTAC |
|  | **118_pluxI_R** | GTCAGACCCCGTAGACTAGTAACAACTTATATCGTATGGG |
|  | **119_luxAf_F** | CTCAAGCTATGCATCAAGCTTGGTACCTCAGGAGGGGCAAATATGAATAAATG |
|  | **120_luxAf_R** | GATGCCTCTAGATTAATTAATTAAGCGGCCGCCATTCGCCAT |
|  | **162_C55_Sal_F** | AGCAATCACCTATGAACTGTCGACCGGCTTTGGCGTATGG |
|  | **163_RBS_R** | CATTTATGTTTTTCATGGGTACATTTGCCCCTCCTGAGCT |
|  | **122_luxI_R** | ATATTTGCCCCTCCTGAGGTACctattttccctataatatacttagtatttaaaataaattaatg |
|  | **136_luxR_F** | ccAACCTTACCAGAGGGCGCCcttcctggttcagagcctcatatcca |
|  | **147_luxR_R** | ccAACCTTACCAGAGGGCGCCacttacgtacttaatttttaaagtatgggcaatcaat |
|  | **148_luxI_R** | tcattaaaacggtaatggattgacatttgattctaataaattggatttttgtcac |
|  | **149_luxI_R2** | cctattgtttgtcgcaagttttgcgtgttatatatcattaaaacggtaatggattgac |
|  | **150_luxR_F** | cttgcgacaaacaataggtaaggataaagagatgggtatgaaaaacataaatgccgacg |
|  | **161_luxI_Kpn_R** | CCCCTCCTGAGGTACCgtcataccaacctcccttgcgt |
|  | **186_yhaJ_SphI_F** | AGCAATCACCTATGAACTGGCATGCACCATCCGTAGT |
|  | **187_yhaJ_SalI_R** | ATACGCCAAAGCCGGGTCGACTTCTGGCAGCAAT |
